# Greater Calcium Intake is Associated with Better Bone Health Measured by Quantitative Ultrasound of the Phalanges in Pediatric Patients Treated with Anticonvulsant Drugs

**DOI:** 10.3390/nu7125517

**Published:** 2015-12-01

**Authors:** Vicente Vera, Jose M. Moran, Patricia Barros, Maria L. Canal-Macias, Rafael Guerrero-Bonmatty, Carmen Costa-Fernandez, Jesus M. Lavado-Garcia, Raul Roncero-Martin, Juan D. Pedrera-Zamorano

**Affiliations:** Metabolic Bone Disease Research Group, School of Nursing and Occupational Therapy, University of Extremadura, Cáceres 10003, Spain; viventevera@odon.ucm.es (V.V.); jmmorang@unex.es (J.M.M.); pbarrosg@yahoo.es (P.B.); luzcanal@unex.es (M.L.C.-M.); rafaelbonmatty@unex.es (R.G.-B.); ccosta@unex.es (C.C.-F.); jmlavado@unex.es (J.M.L.-G.); rroncero@unex.es (R.R.-M.)

**Keywords:** antiepileptic therapy, bone health, quantitative ultrasound, children

## Abstract

We aimed to investigate and compare the effects of chronic antiepileptic therapy on bone health in pediatric patients using quantitative ultrasound of the phalanges (QUS) and controlling for potential confounding factors, particularly nutrient intake. The amplitude-dependent speed of sound (Ad-SoS) was measured in 33 epileptic children and 32 healthy children aged 6.5 ± 3.1 and 6.3 ± 1.1 (mean ± SD) years, respectively. There were no significant differences in the demographics such as age, weight and height between epileptic children and the control group children. None of the children in the epileptic or the treatment group were found to have a vitamin D deficiency. There were no significant differences in laboratory tests between groups. Lower QUS figures were found in the epileptic children (*p =* 0.001). After further adjustment for potential confounders such age, height, weight, calcium intake, vitamin D intake, physical activity and sex, the differences remained significant (*p <* 0.001). After further classification of the participants based on the tertile of calcium intake, no significant differences were found between patients and healthy controls in the greatest tertile of calcium intake (*p =* 0.217). We conclude that anticonvulsant therapy using valproate may lead to low bone mass in children and that an adequate intake of calcium might counteract such deleterious effects.

## 1. Introduction

The association of skeletal abnormalities with chronic antiepileptic therapy (AED) was first described approximately three decades ago, and most of the available data are from adults [1]. In fact, AEDs were associated with several abnormalities in calcium metabolism, including hypocalcemia, hypophosphatemia, elevated levels of serum alkaline phosphatase and serum parathyroid hormone, reduced serum levels of biologically active vitamin D metabolites, radiologic evidence of rickets, and histologic evidence of osteomalacia [2,3,4].

The majority of these studies were performed in adult patients, and data from children receiving AEDs are not fully clear. Particularly, only few studies investigated the effects of AEDs on the bone health of children in quantitative way such as a plain film of X-ray, dual-energy X-ray absorptiometry (DEXA), and histology. However, a no radiation and non-invasive method, such as quantitative ultrasound (QUS) is better because of the safety concerns of these tests and the variable resolution [5,6].

Phalangeal QUS can be measured by two parameters the amplitud dependent speed of sound (Ad-SoS) that reflects the velocity, partly amplitude-dependent, at which the ultrasound wave cross the bone and soft tissue [7] and the bone transmission time BTT that reflects the transmission time of the ultrasound wave through the bone tissue [8]. Ad-SoS is strictly dependent on the thickness of the soft tissue surrounding the phalanges. Due to that dependence, a reduction in the speed of the utrasound wave can be misinterpreted as a reduction in the quality of the bone, while it is likely just an artifact. Alternatively, the second parameter BTT is independent of the soft tissue surrounding the phalanges so both parameters complement each other. 

Therefore, in this study, we investigated and compared the effects of AEDs on bone health in pediatric patients using the QUS of the phalanges and by controlling for potential confounding factors, particularly nutrient intake.

## 2. Materials and Methods 

A total of 33 epileptic children undergoing AED and 32 healthy children were included in this cross-sectional study; the groups had a mean age of 6.5 ± 3.1 and 6.3 ± 1.1 years, respectively. Participants were recruited from a clinical convenience sample. All of the subjects resided in the health district of the province of Caceres, Spain. The children’s parents provided their written informed consent. The Office for Protection against Research Risks of the University of Extremadura approved the study.

Height measurements were made using a Harpender stadiometer with the mandibular plane parallel to the floor, and patients were weighed on a biomedical balance. Both measurements were made with subjects wearing pajamas and without shoes. We calculated BMI (weight/height2) and used the WHO referent to estimate age- and sex-specific BMI z-scores [9]. Food was quantified using a dietetic scale, measuring cups, cans, small bottles and spoons on the basis of current 7-day dietary records as in previous studies [10,11]. The questionnaire used was self-reported, and the person completing the interview was blinded to the research question and hypothesis.

### 2.1. Ultrasound Studies

As in previous studies [12,13,14], all of the children underwent an ultrasound study of the second to fifth proximal phalanges of the non-dominant hand, and the mean of all the measurements was calculate. The QUS study was performed using a model DBM Sonic Bone Profiler (Igea, Capri, Italy) equipped with a caliper that closes tangentially on the phalanx and measures the amplitude-dependent speed of sound (Ad-SoS) in meters per second through the phalanx. Positioning and repositioning the instrument is easy because it uses the prominences of the lower phalangeal epiphysis as a reference; the clip is placed just behind the prominences. At each measuring session, the reference speed of the patient's soft tissue is measured by applying the probes to the soft tissue area between the base of the thumb and the index finger. The device automatically calculates the average AD-SOS of the four measurements, corrected for the presence of soft tissue. All procedures of the QUS were conducted by trained nurses working to standard protocols. The instrument transmits at a frequency of 1.2 MHz with 22 W of power. The instrument’s precision was determined from three measurements in eight subjects at time intervals not exceeding 21 days. The coefficient of variation was 0.77%. The inter-observer coefficient of variation was 1.1%.

### 2.2. Analytical Studies

No exercise was permitted 24 h before the investigation. The hematological and biochemical studies were performed on blood samples taken during a fasting state at 8:00 a.m.

The blood samples were centrifuged, and the serum was stored at −20 °C until analysis. All of the samples were analyzed in the same assay to eliminate inter-assay variation. Assay reproducibility was determined by assaying four samples five times in five different runs. The plasma calcium (Ca), phosphorous (P), parathyroid hormone (PTH), alkaline phosphatase (ALP), bone alkaline phosphatase (BAP) and vitamin D levels of the patient and control groups were measured. 

### 2.3. Physical Activity Assessment

We assessed participants’ physical activity status on the basis of their answer to the following question: “How much do you exercise or strain yourself physically in your leisure time?” The response categories were (1) nonambulation; (2) sedentary (reading, watching television); (3) moderate (walking, cycling, and exercising in other ways for at least 4 h per week); (4) active (fitness-improving sport at least three times per week); and (5) competitive sport [15,16]. None of the participants selected the competitive sport response. 

### 2.4. Statistical Studies

Normal distribution and homogeneity of variances were assessed using the Kolmogorov–Smirnov and Levene tests, respectively. Unpaired t test and analysis of variance (ANOVA) (followed by Bonferroni’s post-hoc test) were used when appropriate to examine relationships between variables. Analysis of co-variance (ANCOVA) was used to compare variables adjusted for the co-variants age, height, weight, vitamin D/calcium intake, physical activity and sex. A few variables were not normally distributed, and a bootstrap procedure was applied to the ANCOVA test. In that case, comparisons were performed with a non-parametric Mann–Whitney U test or a Kruskal Wallis test. Non-parametric correlation (Spearman's rho) was used to correlate QUS with the studied variables. A minimum *P*-value of <0.05 was the necessary condition for statistical significance. Stepwise multiple linear regression analyses were executed to estimate the linear relationship between dependent variables (QUS) and various independent variables. These studies were performed using SPSS 19.0(SPSS Inc., Chicago, IL, USA).

## 3. Results

Thirty-three epileptic children, 13 girls and 20 boys, aged 6.5 ± 3.1 years were enrolled in this study. The 32 healthy control children comprised 14 girls and 18 boys aged 6.3 ± 1.1 years (mean ± SD). There were no significant differences in the demographics such as age, weight and height between the epileptic children and the control group. The demographic data are shown in Table 1. There were no significant differences in serum 25-hydroxyvitamin D, phosphorous, parathyroid hormone, alkaline phosphatase, bone alkaline phosphatase and calcium levels between the groups studied (Table 1).

**Table 1:** Demographics and laboratory of patients and control group.

	Patients (*n =* 33)	Controls (*n =* 32)	*p*-Value
Age (years)	6.5 ± 3.1	6.3 ± 1.1	0.679
Gender (female/male)	13/20	14/18	
Weight (kg)	28.3 ± 16.3	26.7 ± 9.3	0.599
Heigth (m)	1.20 ± 0.24	1.24 ± 0.06	0.428
BMI Z-score	0.71 ± 1.35	0.35 ± 1.26	0.179
Ca (mg/dL)	9.49 ± 1.39	9.80 ± 1.10	0.447
P (mg/dL)	5.26 ± 0.54	5.27 ± 0.35	0.952
PTH (pg/mL)	34.49 ± 18.60	31.10 ± 14.96	0.846
25-OH-Vitamin D (µg/L)	46.36 ± 14.70	46.87 ± 15.09	0.672
1-25-OH-Vitamin D (µg/L)	33.43 ± 8.82	33.03 ± 7.81	0.846
ALP (U/L)	188.9 ± 73.6	195.1 ± 84.0	0.846
BAP (U/L)	80.48 ± 47.97	85.13 ± 52.81	0.416

In the treatment group, 24 of the 33 children were diagnosed with epilepsy, and 9 of the 33 were diagnosed with cerebral palsy. The mean duration of antiepileptic drug therapy was 45 *±* 29 months (mean *±* SD). The polypharmacy split of antiepileptic drugs used was 24 (72.7%) on monotherapy with VPA and 9 (27.3%) on polytherapy with other drugs (Table 2). None of the children in the epileptic group or in the treatment group were found to have a vitamin D deficiency.

**Table 2:** Clinical characteristics of the treatment group.

*Clinical Data*	Value
Epilepsy, *n* (%)	24 (72.7)
Cerebral palsy, *n* (%)	9 (27.3)
Deambulation, (negative/positive)	7/26
Duration of AED in months, (mean ± SD) (range)	45 ± 26 (1–120)
*Antiepileptic Drug Therapy, n (%)*	
Monotherapy with VPA	24 (73)
Polytherapy	
2 drugs ^1^	6 (18)
3 drugs ^2^	1 (3)
4 drugs ^3^	1 (3)
6 drugs ^4^	1 (3)

^1^ (VPA+carbamazepine, *n =* 3), (VPA+phenobarbital, *n =* 1), (VPA+lamotrigine, *n =* 1), (VPA+topiramate, *n =* 1); ^2^ (VPA+vigabatrine+topiramente); ^3^ (VPA, phenytoin, lamotrigine, carbamazepine); ^4^ (VPA, phenobarbital, phenytoin, vigabatrine, lamotrigine, topiramate).

Data from the nutrient intake study are shown in Table 3. There were no differences between the two groups in the intake of phosphorous, iron, zinc, fat, carbohydrates, kilocalories, iodine, magnesium, fluor, cupper, selenium and folic acid (Table 3, *p >* 0.05 in all cases).

There were differences in the intake of proteins (*p =* 0.049) and major food groups. Significant differences were found in the daily intake of meat, cereals and fruits (*p <* 0.05 in both cases) between the two groups (Table 3).

**Table 3:** Nutrient intake study.

	Patients (*n =* 33)	Controls (*n =* 32)	*p*-Value
*Nutrients*			
Proteins (g/day)	101.36 ± 58.87	106.86 ± 28.07	0.049
Carbohydrates (g/day)	228.4 ± 85.8	241.9 ± 57.7	0.215
Fat (g/day)	102.31 ± 63.10	107.65 ± 27.41	0.232
Kilocalories (kcal/day)	2202.2 ± 996.6	2330.4 ± 533.4	0.177
Calcium (mg/day)	1850 ± 777	1989 ± 553	0.198
Phosporous (mg/day)	2164 ± 1013	2215 ± 638	0.259
Iron (mg/day)	27.49 ± 16.00	27.82 ± 11.08	0.520
Zinc (mg/day)	10.50 ± 7.54	10.43 ± 3.40	0.181
Magnesium (mg/day)	425.0 ± 192.6	417.1 ± 155.4	0.618
Fluor (µg/day)	665 ± 307	702 ± 259	0.435
Cupper (mg/day)	1.28 ± 1.4	0.95 ± 0.46	0.844
Iodine (ng/day)	281 ± 258	303 ± 316	0.922
Selenium (µg/day)	92.0 ± 57.2	92.6 ± 25.1	0.546
Folic acid (µg/day)	179.1 ± 81.2	184.2 ± 62.1	0.474
Vitamin D (µg/day)	6.26 ± 7.14	6.88 ± 5.02	0.201
Vitamin E (mg/day)	2.34 ± 0.866	2.65 ± 0.81	0.072
*Major foods (servings/week)*			
Fats	10 ± 4	8 ± 2	0.190
Dairy	17 ± 5	18 ± 4	0.374
Meat	12 ± 13	12 ± 5	0.028
Fish	3 ± 2	4 ± 2	0.071
Cereals	15 ± 6	19 ± 5	0.044
Fruits	13 ± 11	16 ± 8	0.044
Vegetables	7 ± 8	8 ± 5	0.252
Carbohydrates	9 ± 6	12 ± 5	0.057
Eggs	2 ± 1	2 ± 1	0.446

Quantitative ultrasound data were analyzed and shown in Table 4. There were significant differences between the two studied groups in that the control group had greater quantitative ultrasound values than the treatment group (Table 4) (*p =* 0.003). After further adjustment for potential confounders such age, height, weight, calcium intake, vitamin D intake, physical activity and sex, the differences remained significant (*p <* 0.0001) (Table 4).

**Table 4:** Quantitative ultrasound of the phalanges study.

	Patients (*n =* 33)	Controls (*n =* 32)	*p*-Value
Ad-SoS (m/s)	1850.06 ± 81.31	1903.00 ± 36.30	0.003
Ad-SoS Z-score	−0.76 ± 1.54	0.42 ± 0.071	<0.0001
*Adjusted Ad-SoS (m/s)*	1850.82 ± 10.15	1849.18 ± 9.50	<0.0001 ^1^
BTT µsec	0.74 ± 0.21	0.93 ± 0.28	0.022
BTT Z-score	−0.23 ± 1.10	0.88 ± 1.15	0.007
*Adjusted BTT (µsec)*	0.69 ± 0.58	0.96 ± 0.04	0.016^1^

^1^ After further adjustment by potential confounding factors (age, height, weight, calcium intake, vitamin D intake, physical activity and sex) (Bootstrap ANCOVA test).

There were positive bivariate correlations in the treatment group between QUS and weight (*r =* 0.659; *p <* 0.0001), age (*r =* 0.596; *p =* 0.001), height (*r =* 0.774; *p <* 0.0001) and the intake of calcium (*r =* 0.634; *p =* 0.009), phosphorous (*r =* 0.573; *p <* 0.001), iron (*r =* 0.592; *p <* 0.001), proteins (*r =* 0.499; *R =* 0.003), fat (*r =* 0.609; *p <* 0.0001), carbohydrates (*r =* 0.400; *p =* 0.021), kilocalories (*r =* 0.572; *R =* 0.001), magnesium (*r =* 0.643; *p <* 0.001) and selenium (*r =* 0.549; *p <* 0.001). A negative correlation was found with iodine intake (*r =* −0.463; *p =* 0.007). Duration of AED did not correlate with Ad-SoS (*p =* 0.346) or BTT (*p =* 0.920) measurements.

In the control group, a positive correlation was found with age (*r =* 0.592; *p <* 0.001), height (*r =* 0.649; *p <* 0.001) and weight (*r =* 0.389; *p =* 0.028).

After further adjustment for potential confounders (age, eight weight, calcium intake, vitamin D intake, physical activity and sex) calcium intake and phosphorous intake remained as positive correlations (*r =* 0.557; *p <* 0.006 and *r =* 0.447; *r =* 0.032, respectively) in the patients group. In the control group, none of the correlations remained after further adjustment for potential confounding factors. 

Participants were further classified based on their tertile of calcium intake (Table 5). Significant differences were found in the treatment group with respect to the QUS figures (*p <* 0.001). Between the lower and the higher tertile of calcium intake, the QUS was significantly greater in the group with greater calcium intake (*p <* 0.001) whereas no differences were found between the lower and the middle tertiles (*p >* 0.005). In the control group, there were no differences in the QUS figures between the groups when the groups were divided into tertile based on calcium intake (*p =* 0.927). 

**Table 5:** Quantitative ultrasound (QUS) figures based in the calcium intake tertile.

Tertile	Patients (*n =* 33)	*p*-Value	Control (*n =* 32)	*p*-Value
Lower	1815.45 ± 45.65		1906.45 ± 28.37	
Medium	1805.36 ± 74.90	0.699 ^3^	1900.45 ± 41.45	0.606 ^4^
Higher	1929.36 ± 55.03	<0.001 ^1^	1902.00 ± 41.37	0.512 ^2^

^1^ Higher *vs.* lower tertile*;* ^2^ Higher *vs.* lower tertile*;* ^3^ Medium *vs.* lower tertile*;* ^4^ Medium *vs.* lower tertile.

We further compared the tertiles between groups. There were differences between participants in the lower and middle tertiles of calcium intake (*p <* 0.0001 and *p =* 0.009, respectively) whereas no significant differences between groups were found in the higher tertile of calcium intake (*p =* 0.181) (Table 6). After further adjustment for potential confounders (age, height, weight, vitamin D intake, physical activity and sex) differences remained significant between the lower and middle tertiles (*p =* 0.001 and *p <* 0.0001, respectively). No significant differences were found between patients and healthy controls in the higher tertile of calcium intake (*p =* 0.217) (Table 6).

**Table 6:** QUS figures based in the calcium intake tertile and between groups.

Tertile		Patients (*n =* 33)	Control (*n =* 32)	*p*-Value
Lower	*Adjusted ^1^*	1815.45 ± 45.65	1906.45 ± 28.37	<0.0001
		1817.31 ± 12.63	1908.01 ± 11.03	0.001
Medium	*Adjusted ^1^*	1805.36 ± 74.90	1900.45 ± 41.45	0.009
		1835.41 ± 19.34	1862.34 ± 17.93	<0.0001
Higher	*Adjusted ^1^*	1929.36 ± 55.03	1902.00 ± 41.37	0.181
		1919.70 ± 22.62	1897.96 ± 22.62	0.489

^1^ After further adjustment by potential confounding factors (age, height, weight, calcium intake, vitamin D intake, physical activity and sex) (Bootstrap ANCOVA test).

Similarly, participants were classified based on the tertile of vitamin D intake. Significant differences between groups were observed in the lower (*p =* 0.037), the middle (*p =* 0.029) and the higher tertile of vitamin D intake (*p =* 0.019) (Table 7) when adjusted by age, height, weight, calcium D intake, physical activity and sex. The QUS figures were always greater in the control group *vs.* the treatment group.

**Table 7:** QUS figures based in the vitamin D intake tertile and between groups.

Tertile		Patients (*n =* 33)	Control (*n =* 32)	*p*-Value
Lower	*Adjusted ^1^*	1855.66 ± 18.48	1903.11 ± 16.51	0.037
Medium	*Adjusted ^1^*	1900.88 ± 28.39	1850.61 ± 28.39	0.029
Higher	*Adjusted ^1^*	1824.54 ± 23.50	1893.07 ± 21.43	0.019

^1^ After further adjustment by potential confounding factors (age, height, weight, calcium intake, calcium intake, physical activity and sex) (Bootstrap ANCOVA test).

The main determinants of QUS were examined by multiple regression analysis. For the QUS, the variables used in the model as independent predictors were the following: duration of AED (patients group only), weight, height, age and daily intake of vitamin D, vitamin E, calcium, phosphorous, iron, zinc, proteins, fat, carbohydrates, kilocalories, iodine, magnesium, cupper, selenium and folic acid. Height and calcium intake were the variables that contributed significantly to QUS variance (β = 0.052(0.013), *p =* 0.001 for calcium intake; β = 208.57 (46.08), *p <* 0.0001 for heigth) in the treatment group. In the control group, height was the only positive predictor (β = 293.38(81.10), *p =* 0.001).

## 4. Discussion

In the current study, there were significant reductions in QUS figures by the means of Ad-SoS in the patients treated with VPA compared with the control group. However, there were no significant differences in the several markers of bone metabolism tested. In support of our findings, a number of previous studies have found similar results measuring bone mineral density in pediatric patients using dual-energy X-ray absorptiometry (DXA). Kafali and colleagues [6] found decreased bone mineralization in children with epilepsy that were treated with VPA even though treatment was for a rather short time. Similarly, Sheth and colleagues [17] observed that valproate monotherapy but not carbamazepine therapy significantly reduced axial and appendicular bone mineral density in children with idiopathic epilepsy and that the treatment might also increase the patient’s risk of osteoporotic fractures. However, Chou *et al.* observed that in 42 children with uncomplicated epilepsy who were treated with either carbamazepine and VPA monotherapy for more than 6 months, receiving carbarmazepine monotherapy had increased the frequency of lower bone density compared with children receiving VPA monotherapy [18]. Conversely, recent data in a cohort of 32 pediatric patients (aged 8.6 ± 4.6 years) showed that there was no considerable bone loss in patients receiving long-term anticonvulsant medication, even after two years of treatment [19]. 

Phalangeal QUS may be a useful method to assess bone quality and fracture risk in children and adolescents with bone and mineral disorders [12,20,21]. Using QUS, similar results as those previously described using DXA have been found in adults [13,14]. Indeed, QUS has been proposed to have a putative role in monitoring bone loss in epileptic patients and in guiding suitable preventive therapy [14]. However, there is a lack in studies with respect to addressing the use of QUS techniques in children receiving anticonvulsant therapy [22]. 

Although multiple mechanisms have been postulated to support the association between antiepileptic drugs and bone disease, including histologic, radiographic, and biochemical evidence [23,24], no single mechanism explains all the findings. There is, however, a mechanism that has been proposed to be a key event in the association between anticonvulsants and the lack of bone mass: antiepileptic drugs may interfere with intestinal absorption of calcium, which can lead to hypocalcemia and feedback hypersecretion of PTH [17,23,24,25]. We have not observed differences in the plasma levels of calcium and PTH between patients and controls in a manner similar to previously described results [6,26,27]. Our data support previous observations from Tsukahara and colleagues, as they described that dietary calcium intake in the osteopenic patients was significantly less than the dietary calcium intake of the non-osteopenic patients [27]. These results indicate that simultaneous supplementation with oral calcium and 25-OHvit D is effective in preventing the development of biochemical changes that indicate predisposition to the development of rickets or osteomalacia in children [28].

In pediatric patients with cerebral palsy it has been described that QUS measurements might be altered due to hand griposis [29]. Thus, if only Ad-SoS is employed to estimate bone strength as it is strictly dependent on the thickness of the soft tissue surrounding the phalanges, Ad-SoS measurements could lead to an underestimation of bone quality in obese subjects and to an apparently better bone status in thin individuals. The soft tissue thickness effect is well known and described [30,31,32,33]. We have not found differences in the BMI Z-score of patients and controls and both Ad-SoS and BTT measurements were higher in the control group. As BTT is largely independent of soft tissue bias in the phalanges of the hand [23] and low values of Ad-SoS are associated with a reduced BMD assessed by DXA [20,34] we think that our data based in the QUS figures confirm a worst bone quality in pediatric patients undergoing AED.

Recent data have also indicated that in children with epilepsy receiving antiepileptic drugs, one of the main determinants of normal BMD is the absence of vitamin D deficiency [35]. We did not observe any case of vitamin D deficiency, but the QUS technique was able to detect bone mass differences between the groups. Most patients in our study were ambulant and the sunlight exposure in our area is one of the greatest in Spain [36], so vitamin D deficiency could not be a key determinant of bone mass in Spanish children. 

We recognize several limitations in our study: (i) the main limitation of our study was its relatively small population; (ii) the second limitation was that this was a cross-sectional study, and basal QUS values were not available; (iii) the third limitation was a lack of control of sun exposure that might have led to an underestimation of the potential role of vitamin D in the results presented. 

Conversely, our study has several strengths: (i) our study added to the limited knowledge with respect to the putative use of QUS techniques in pediatric populations undergoing anticonvulsant therapy; (ii) we controlled our analysis using a number of confounding factors that have not been addressed in previous studies, particularly the physical activity and dietary factors of the patients.

## 5. Conclusions 

In conclusion, our study shows that anticonvulsant therapy with VPA may lead to low bone mass in children and that an adequate intake of calcium might counteract such deleterious effects. In our patient population, we have shown that changes in bone health may be adequately addressed through the use of quantitative ultrasound of the phalanges, which is a technique that is portable and uses ionizing radiation-free equipment. Additional research is needed to clarify the effects of calcium and/or dairy products on low bone mass detected in pediatric patients under AED treatment and to elucidate potential biological mechanisms.
